# Recurrent copy number variations in the human fungal pathogen *Candida parapsilosis*

**DOI:** 10.1128/mbio.00713-23

**Published:** 2023-10-03

**Authors:** Anna Selmecki

**Affiliations:** 1 Department of Microbiology and Immunology, University of Minnesota Medical School, Minneapolis, Minnesota, USA; Tel Aviv University, Tel Aviv, Israel

**Keywords:** copy number variation, *Candida parapsilosis*, antifungal drug resistance, experimental evolution

## Abstract

*Candida parapsilosis* is an opportunistic fungal pathogen with increasing incidence in hospital settings worldwide; however, we lack a comprehensive understanding of the mechanisms promoting its virulence and drug resistance. Bergin et al. systematically quantify the frequency and effect of copy number variation (CNV) across 170 diverse clinical and environmental isolates of *C. parapsilosis* (Bergin SA, Zhao F, Ryan AP, Müller CA, Nieduszynski CA, Zhai B, Rolling T, Hohl TM, Morio F, Scully J, Wolfe KH, Butler G, 2022, mBio, https://doi.org/10.1128/mbio.01777-22). Using a combination of both short- and long-read whole genome sequencing techniques, they determine the structure and copy number of two CNVs that arose recurrently throughout the evolution of these isolates. Each CNV predominantly amplifies one coding sequence (*ARR3* or *RTA3*); however, the amplitude and recombination breakpoints are variable across the isolates. Amplification of *RTA3* correlates with drug resistance and deletion causes drug susceptibility. This study highlights the need for further research into the mechanisms and dynamics of CNV formation and the impact of these CNVs on virulence and drug resistance across diverse fungal pathogens.

## COMMENTARY

Invasive fungal infections caused by *Candida* species are an important cause of morbidity and mortality worldwide. These fungi are commensal to human skin and mucosal membranes but can cause life-threatening infections in immunocompromised individuals and are often associated with central venous catheterization and other indwelling devices. While *Candida albicans* is the most common pathogen associated with invasive candidiasis, non-albicans *Candida* species such as *C. glabrata*, *C. parapsilosis*, *C. tropicalis*, *C. krusei*, and *C. auris* have increased in incidence in hospital settings worldwide (1, 2). Non-albicans *Candida* species are often resistant to commonly used antifungal drugs and are associated with higher mortality rates. Therefore, understanding the epidemiology, pathogenesis, and drug resistance of non-albicans *Candida* species is of utmost importance for the prevention and treatment of *Candida* infections.

*C. parapsilosis* was first identified in 1928, is widely distributed in the environment, and is isolated from many non-human sources including soil, insects, and animals (3 – 5). Since 1990, *C. parapsilosis* has undergone the largest increase in incidence of any *Candida* species worldwide; however, these infections exhibit regional differences in frequency and distribution [reviewed in reference (6)]. *C. parapsilosis* is the second-most isolated *Candida* species in South America, Southern Europe, India, and Pakistan and even outranks *C. albicans* as the most frequently isolated *Candida* species in Venezuela and Colombia (7 – 10). In addition to disproportionate effects in certain regions, *C. parapsilosis* causes a disproportionate amount of disease in neonates (11, 12). Compared to *C. albicans*, we know relatively little about the molecular mechanisms that have enabled *C. parapsilosis* to achieve this prominence in hospital settings.

*C. parapsilosis* is a diploid organism, yet it exhibits a remarkably low level of heterozygosity (~1 SNP every 15,553 bp) compared to the highly heterozygous diploid pathogen *C. albicans* (~1 SNP per 330 bp) (13 – 16). Early theories suggested that this low level of heterozygosity in clinical isolates was due to a global expansion of a virulent clone (a very recent population bottleneck); however, whole-genome sequencing has revealed genomic signatures that argue against a clonal expansion. In 2013, Pryszcz et al. performed the first comparative analysis of four *C. parapsilosis* genomes from two environmental isolates (Italy and USA) and two clinical isolates (Germany and USA) (14). They validated the low levels of heterozygosity within isolates but also reported high levels of genomic variability between isolates, including single-nucleotide variants (SNVs), copy number variations (CNVs), and genomic rearrangements. This comprehensive comparative genomics analysis of four isolates provided the first evidence for recombination in *C. parapsilosis*. Specifically, they found that SNVs are not homogeneously distributed along the chromosomes but occur in SNV clusters (14). Such clusters have a low probability of arising via independent spontaneous point mutations and may have arisen via recombination between strains. Additionally, in just these four *C. parapsilosis* genomes, there is clear evidence for intrachromosomal recombination events between repeat sequences that promoted CNV formation, including gene deletion, gene amplification, and in-frame gene fusion events (14). These data suggested that CNVs may be important to the evolution of *C. parapsilosis*, but the frequency and effect of CNVs on virulence phenotypes were not known.

CNVs are an important source of genetic diversity with clinical relevance in pathogenic fungi and human disease. CNVs can impact the expression of many genes simultaneously and provide rapid adaptation to stress, including nutrient limitation, elevated temperature, and antifungal drug stress. In *Saccharomyces cerevisiae*, amplification of genes encoding proteins involved in nutrient acquisition (*HXT6/7*, *SUL1*, *GAP1*, *DUR3*, *CUP1*, etc.) is common in nutrient poor environments, including carbon-, salt-, phosphate-, glutamine-, and urea-limited environments (17 – 22). In *C. albicans*, recurrent amplification of an entire chromosome arm in an isochromosome structure doubles the DNA copy number of two genes (*ERG11* and *TAC1*) that are necessary and sufficient to cause multi-azole drug resistance (23 – 25). Additionally, some CNVs are rapidly expandable in the presence of selection and reversible in the absence of selection, including some high copy number CNVs that arise during adaptation to azole drugs (26 – 28). Despite their importance, CNVs are understudied relative to SNVs, in part because of the inherent difficulties in detecting low-frequency CNVs within populations of heterogeneous cells and because CNVs often occur at/near repetitive genomic sequences with poor mappability. Indeed, errors in DNA replication and/or repair at repetitive sequences frequently promote the formation of high copy number CNVs even in the absence of selection (29, 30). Overall, these studies highlight the importance of CNVs in the evolution of fungi and emphasize that further research is needed to fully understand the molecular mechanisms underlying CNV formation.

Bergin et al. performed the largest phylogenetic analysis of *C. parapsilosis* isolates to date, incorporating 170 new and published genomes that include predominantly human isolates collected from two hospitals (Memorial Sloan Kettering Cancer Center in New York and the Centre Hospitalier Universitaire de Nantes, France) with some additional environmental isolates (31). The genome-wide SNP analysis approach powerfully shows that there is little evidence for geographical clustering of *C. parapsilosis*. For example, two clinical isolates from New York and France cluster closely with an Irish soil isolate, highlighting the wide distribution and global nature of *C. parapsilosis* as a human pathogen.

Considering the relatedness of their isolates, Bergin et al. then performed a systematic and comprehensive analysis of CNVs across the 170 isolates. They identified examples of both large and small CNVs, including whole chromosome aneuploidy (trisomy) in nine of the isolates and many examples of CNVs in the subtelomeric regions. Excluding the subtelomeres, they identified 16 examples of large CNVs, >10 kb in size. These large CNVs included both amplification and deletions of large genomic regions (ranging from ~10 to 150 kb). Additionally, three large CNVs had complex stair-step structures, where a highly amplified central core region was surrounded by two regions with lower copy numbers that were themselves flanked by inverted repeat sequences (1–5.4 kb in length) (31). These CNVs are strikingly similar to the complex, stair-step CNVs that arise in the diploid *C. albicans* during *in vitro* adaptation to azole drugs (28). The complex, stair-step CNVs in *C. parapsilosis* are unique in that they are the first to be described in any *Candida* isolate obtained from human patients. It would be valuable to know if these patients were treated with azole antifungal drugs and if these large CNVs promote azole resistance in the patient.

A single *C. parapsilosis* clinical isolate (FM07 from CHU de Nantes) carried almost all (13/16) of the large CNVs >10 kb (31). This genetic background may have an increased mutation rate or errors in DNA repair that promote CNV formation. In *C. albicans*, some genetic backgrounds have increased frequencies of complex CNVs (25), and mutator genotypes can rapidly acquire large CNVs in other critical fungal pathogens like *C. auris* (32) and *Cryptococcous neoformans* (33). Further comparative genomics between isolates with high and low CNV frequencies will help identify the molecular mechanisms involved in CNV formation in *C. parapsilosis* and other pathogenic fungi.

Bergin et al. then determined the structure and copy number of ~167 small CNVs (<10 kb in size) in the 170 *C*. *parapsilosis* isolates. Two small CNVs arose multiple times and predominantly amplified a single coding sequence, *ARR3* or *RTA3*. Importantly, the amplitude and the borders of the recombination breakpoints are highly variable across the isolates and support that these small CNVs arose through multiple independent events.

The first small CNV encompasses the ortholog of *S. cerevisiae Arr3*, an arsenate transporter (*CPAR2_601050*). Amplification of *ARR3* was first described by Pryszcz et al. in four isolates of *C. parapsilosis*, each having unique CNV breakpoints and unique copy number amplitude (~6 to 10 copies) (14). Bergin et al. add an additional 44 clinical isolates and 2 environmental isolates with an *ARR3* CNV, comprising 8 distinct CNV breakpoints with copy numbers ranging from 3 to 23 copies. Excitingly, their characterization of several of these unique *ARR3* CNVs with long-read sequencing provides the structure of the entire tandem array of repeating sequences in a single sequencing read. Utilizing their new strain phylogeny, they observed that the eight distinct CNV breakpoint genotypes arose independently throughout the evolution of these isolates. Amplification of *ARR3* copy number correlates with arsenate resistance in other soil fungi, including *S. cerevisiae*, *S. paradoxus,* and *C. neoformans var. grubii* (34, 35). These studies suggest that amplification of the *ARR3* locus has an important physiological role in *C. parapsilosis*, likely for survival in a high-arsenate soil environment. However, an additional role in pathogenesis cannot be excluded.

The second small CNV that occurred in multiple *C. parapsilosis* isolates encompasses the ortholog of *S. cerevisiae RTA3* (*CPAR2_104610*), encoding a putative phosphatidylcholine floppase. Amplification of *RTA3* was detected in 104/170 isolates with 16 different CNV patterns, each with unique copy number breakpoints. Amplification of the *RTA3* coding sequence was shown previously to correlate with increased *RTA3* expression (36, 37), and Bergin et al. identified that amplification of just the *RTA3* promoter region correlates with increased expression of *RTA3*. The *RTA3* copy number was highly variable (4–50 copies per isolate) between isolates, suggesting that the amplification may be due to the formation of extrachromosomal circles. In *S. cerevisiae,* extrachromosomal circles can rapidly increase or decrease in copy number of many genes, including the rDNA and *CUP1* loci (38, 39). To determine if the *C. parapsilosis RTA3* amplifications are intrachromosomal or extrachromosomal, Bergin et al. used long-read sequencing to sequence across five different *RTA3* CNV patterns. In each case, the long reads supported an intrachromosomal amplification of *RTA3*, and the repeat copies of *RTA3* were arranged in a tandem array on the same DNA molecule. Additionally, the long-read sequencing data supported that each homolog in this diploid organism can carry a different *RTA3* copy number.

The recurrent amplification of *RTA3* across many diverse environmental and clinical *C. parapsilosis* isolates suggests that this mutation has an important physiological role. In *C. albicans*, homozygous deletion of *RTA3* increases susceptibility to fluconazole and miltefosine (40, 41). Miltefosine is an anti-parasitic drug primarily used to treat leishmaniasis, but it also has broad-spectrum antifungal activity against *Cryptococcus*, *Aspergillus,* and *Candida,* and in 2021, miltefosine obtained orphan drug designation for invasive Candidiasis by the United States Food and Drug Administration (42, 43). Bergin et al. measured drug susceptibility in the *C. parapsilosis* isolates and found that *RTA3* copy number amplification correlated with miltefosine resistance but not fluconazole resistance. Additionally, homozygous deletion of *RTA3* from the *C. parapsilosis* reference isolate resulted in increased sensitivity to miltefosine. It is tempting to speculate that miltefosine treatment may have been selected for *RTA3* amplification in the *C. parapsilosis* clinical isolates; however, this is unlikely given that this drug was only recently FDA approved.

To determine if *RTA3* CNVs arise during adaptation to miltefosine *in vitro*, Bergin et al. performed experimental evolution in the presence of increasing concentrations of miltefosine with two different progenitor isolates that had just one copy of *RTA3* at each allele. After 26 days, no CNVs of *RTA3* were observed; however, acquisition of *de novo* SNVs in other genes was identified. These SNVs encoded loss of function (LOF) mutations in *CPAR2_102700* and *CPAR2_303950,* which are homologs of the PC flippase genes in *S. cerevisiae*. Impressively, using CRISPR-cas9 mutagenesis, the authors were able to show that LOF of both genes is required for high levels of miltefosine resistance. Overall, these data highlight that there are multiple evolutionary routes to acquire increased resistance to miltefosine, but CNV of *RTA3* did not arise in this *in vitro* experiment. It is likely that selective pressures other than miltefosine are driving recurrent amplification of *RTA3* in *C. parapsilosis*. Alternatively, amplification of *RTA3* may be both rapid and reversible *in vitro,* appearing early during adaptation to miltefosine and lost once *de novo* beneficial point mutations are acquired. The rate and dynamics of CNV formation are difficult to measure; however, fluorescent reporter systems have been used in *S. cerevisiae* to quantify CNV events at the single cell level with flow cytometry (44, 45). More approaches are needed to address the rate and mechanisms of CNV between different isolates, species, and environments.

We recently showed that the concentration of an antifungal drug can dramatically impact the rate and type of CNVs that form during *in vitro* evolution of *C. albicans* (25). Specifically, constant low concentrations of fluconazole (near the MIC_50_ of the original isolates) were selected for CNVs in diverse genetic backgrounds, whereas supra-MIC concentrations of fluconazole were selected for whole chromosome aneuploidy and polyploidy but not CNVs. Ultimately, more work is needed in diverse *Candida* species to identify the mechanism and dynamics driving CNV formation *in vitro* and *in vivo*, both in the presence and absence of antifungal drugs.

In summary, Bergin et al. performed a systematic and elegant bioinformatics analysis of SNVs and CNVs of the largest cohort of *C. parapsilosis* isolates to date. The power of sampling many isolates revealed that DNA amplifications comprising *ARR3* or *RTA3* arose multiple times across the phylogeny of *C. parapsilosis,* and these recurrent patterns of amplification revealed several mechanisms of CNVs formation. In *C. parapsilosis* and other fungal pathogens, adaptation to the host or environmental niche may be promoting CNV formation, and these CNVs may provide unexpected cross-resistance to antifungal drugs. More work is needed to identify the selective forces promoting CNV formation and the effect of those CNVs on drug resistance and virulence in diverse fungal pathogens.

## References

[1] Pfaller MA , Diekema DJ , Turnidge JD , Castanheira M , Jones RN . 2019. Twenty years of the SENTRY antifungal surveillance program: results for Candida species from 1997-2016. Open Forum Infect Dis 6:S79–S94. doi:10.1093/ofid/ofy358 30895218 PMC6419901

[2] Stavrou AA , Lackner M , Lass-Flörl C , Boekhout T . 2019. The changing spectrum of Saccharomycotina yeasts causing candidemia: phylogeny mirrors antifungal susceptibility patterns for azole drugs and amphothericin B. FEMS Yeast Res 19:foz037. doi:10.1093/femsyr/foz037 31158288

[3] Ashford BK . 1928. Certain conditions of the gastro-intestinal tract in porto rico and their relation to tropical sprue. Am J Trop Med Hyg s1-8:507–538. doi:10.4269/ajtmh.1928.s1-8.507

[4] Branco J , Miranda IM , Rodrigues AG . 2023. Candida parapsilosis virulence and antifungal resistance mechanisms: a comprehensive review of key determinants. J Fungi (Basel) 9:80. doi:10.3390/jof9010080 36675901 PMC9862255

[5] Fell JW , Meyer SA . 1967. Systematics of yeast species in the Candida parapsilosis group. Mycopathol Mycol Appl 32:177–193. doi:10.1007/BF02049795 6051845

[6] Trofa D , Gácser A , Nosanchuk JD . 2008. Candida parapsilosis, an emerging fungal pathogen. Clin Microbiol Rev 21:606–625. doi:10.1128/CMR.00013-08 18854483 PMC2570155

[7] Almirante B , Rodríguez D , Cuenca-Estrella M , Almela M , Sanchez F , Ayats J , Alonso-Tarres C , Rodriguez-Tudela JL , Pahissa A . 2006. Epidemiology, risk factors, and prognosis of Candida parapsilosis bloodstream infections: case-control population-based surveillance study of patients in Barcelona, Spain, from 2002 to 2003. J Clin Microbiol 44:1681–1685. doi:10.1128/JCM.44.5.1681-1685.2006 16672393 PMC1479182

[8] Nucci M , Queiroz-Telles F , Alvarado-Matute T , Tiraboschi IN , Cortes J , Zurita J , Guzman-Blanco M , Santolaya ME , Thompson L , Sifuentes-Osornio J , Echevarria JI , Colombo AL , Latin American Invasive Mycosis Network . 2013. Epidemiology of candidemia in Latin America: a laboratory-based survey. PLoS One 8:e59373. doi:10.1371/journal.pone.0059373 23527176 PMC3601956

[9] Guinea J . 2014. Global trends in the distribution of Candida species causing candidemia. Clin Microbiol Infect 20 Suppl 6:5–10. doi:10.1111/1469-0691.12539 24506442

[10] Rodriguez L , Bustamante B , Huaroto L , Agurto C , Illescas R , Ramirez R , Diaz A , Hidalgo J . 2017. A multi-centric study of Candida bloodstream infection in Lima-Callao, Peru: species distribution, antifungal resistance and clinical outcomes. PLoS One 12:e0175172. doi:10.1371/journal.pone.0175172 28419092 PMC5395148

[11] Fridkin SK , Kaufman D , Edwards JR , Shetty S , Horan T , the National Nosocomial Infections Surveillance System Hospitals . 2006. Changing incidence of Candida bloodstream infections among NICU patients in the United States: 1995-2004. Pediatrics 117:1680–1687. doi:10.1542/peds.2005-1996 16651324

[12] Zaoutis T . 2010. Candidemia in children. Curr Med Res Opin 26:1761–1768. doi:10.1185/03007995.2010.487796 20513207

[13] Butler G , Rasmussen MD , Lin MF , Santos MAS , Sakthikumar S , Munro CA , Rheinbay E , Grabherr M , Forche A , Reedy JL , Agrafioti I , Arnaud MB , Bates S , Brown AJP , Brunke S , Costanzo MC , Fitzpatrick DA , de Groot PWJ , Harris D , Hoyer LL , Hube B , Klis FM , Kodira C , Lennard N , Logue ME , Martin R , Neiman AM , Nikolaou E , Quail MA , Quinn J , Santos MC , Schmitzberger FF , Sherlock G , Shah P , Silverstein KAT , Skrzypek MS , Soll D , Staggs R , Stansfield I , Stumpf MPH , Sudbery PE , Srikantha T , Zeng Q , Berman J , Berriman M , Heitman J , Gow NAR , Lorenz MC , Birren BW , Kellis M , Cuomo CA . 2009. Evolution of pathogenicity and sexual reproduction in eight Candida genomes. Nature 459:657–662. doi:10.1038/nature08064 19465905 PMC2834264

[14] Pryszcz LP , Németh T , Gácser A , Gabaldón T . 2013. Unexpected genomic variability in clinical and environmental strains of the pathogenic yeast Candida parapsilosis. Genome Biol Evol 5:2382–2392. doi:10.1093/gbe/evt185 24259314 PMC3879973

[15] Muzzey D , Schwartz K , Weissman JS , Sherlock G . 2013. Assembly of a phased diploid Candida albicans genome facilitates allele-specific measurements and provides a simple model for repeat and Indel structure. Genome Biol 14:R97. doi:10.1186/gb-2013-14-9-r97 24025428 PMC4054093

[16] Hirakawa MP , Martinez DA , Sakthikumar S , Anderson MZ , Berlin A , Gujja S , Zeng Q , Zisson E , Wang JM , Greenberg JM , Berman J , Bennett RJ , Cuomo CA . 2015. Genetic and phenotypic intra-species variation in Candida albicans. Genome Res 25:413–425. doi:10.1101/gr.174623.114 25504520 PMC4352881

[17] Welch JW , Fogel S , Cathala G , Karin M . 1983. Industrial yeasts display tandem gene iteration at the CUP1 region. Mol Cell Biol 3:1353–1361. doi:10.1128/mcb.3.8.1353-1361.1983 6621529 PMC369981

[18] Brown CJ , Todd KM , Rosenzweig RF . 1998. Multiple duplications of yeast hexose transport genes in response to selection in a glucose-limited environment. Mol Biol Evol 15:931–942. doi:10.1093/oxfordjournals.molbev.a026009 9718721

[19] Dunham MJ , Badrane H , Ferea T , Adams J , Brown PO , Rosenzweig F , Botstein D . 2002. Characteristic genome rearrangements in experimental evolution of Saccharomyces cerevisiae. Proc Natl Acad Sci U S A 99:16144–16149. doi:10.1073/pnas.242624799 12446845 PMC138579

[20] Gresham D , Desai MM , Tucker CM , Jenq HT , Pai DA , Ward A , DeSevo CG , Botstein D , Dunham MJ . 2008. The repertoire and dynamics of evolutionary adaptations to controlled nutrient-limited environments in yeast. PLoS Genet 4:e1000303. doi:10.1371/journal.pgen.1000303 19079573 PMC2586090

[21] Payen C , Di Rienzi SC , Ong GT , Pogachar JL , Sanchez JC , Sunshine AB , Raghuraman MK , Brewer BJ , Dunham MJ . 2014. The dynamics of diverse segmental amplifications in populations of Saccharomyces cerevisiae adapting to strong selection. G3 (Bethesda) 4:399–409. doi:10.1534/g3.113.009365 24368781 PMC3962480

[22] Yona AH , Manor YS , Herbst RH , Romano GH , Mitchell A , Kupiec M , Pilpel Y , Dahan O . 2012. Chromosomal duplication is a transient evolutionary solution to stress. Proc Natl Acad Sci U S A 109:21010–21015. doi:10.1073/pnas.1211150109 23197825 PMC3529009

[23] Selmecki A , Forche A , Berman J . 2006. Aneuploidy and isochromosome formation in drug-resistant Candida albicans. Science 313:367–370. doi:10.1126/science.1128242 16857942 PMC1717021

[24] Selmecki A , Gerami-Nejad M , Paulson C , Forche A , Berman J . 2008. An isochromosome confers drug resistance in vivo by amplification of two genes, ERG11 and TAC1. Mol Microbiol 68:624–641. doi:10.1111/j.1365-2958.2008.06176.x 18363649

[25] Todd RT , Soisangwan N , Peters S , Kemp B , Crooks T , Gerstein A , Selmecki A . 2023. Antifungal drug concentration impacts the spectrum of adaptive mutations in Candida albicans. Mol Biol Evol 40:msad009. doi:10.1093/molbev/msad009 36649220 PMC9887641

[26] Mount HO , Revie NM , Todd RT , Anstett K , Collins C , Costanzo M , Boone C , Robbins N , Selmecki A , Cowen LE . 2018. Global analysis of genetic circuitry and adaptive mechanisms enabling resistance to the azole antifungal drugs. PLoS Genet 14:e1007319. doi:10.1371/journal.pgen.1007319 29702647 PMC5922528

[27] Todd RT , Wikoff TD , Forche A , Selmecki A . 2019. Genome plasticity in Candida albicans is driven by long repeat sequences. Elife 8:e45954. doi:10.7554/eLife.45954 31172944 PMC6591007

[28] Todd RT , Selmecki A . 2020. Expandable and reversible copy number amplification drives rapid adaptation to antifungal drugs. Elife 9:e58349. doi:10.7554/eLife.58349 32687060 PMC7371428

[29] Thierry A , Khanna V , Dujon B . 2016. Massive amplification at an unselected locus accompanies complex chromosomal rearrangements in yeast. G3 (Bethesda) 6:1201–1215. doi:10.1534/g3.115.024547 26945028 PMC4856073

[30] Brewer BJ , Payen C , Raghuraman MK , Dunham MJ . 2011. Origin-dependent inverted-repeat amplification: a replication-based model for generating palindromic amplicons. PLoS Genet 7:e1002016. doi:10.1371/journal.pgen.1002016 21437266 PMC3060070

[31] Bergin SA , Zhao F , Ryan AP , Müller CA , Nieduszynski CA , Zhai B , Rolling T , Hohl TM , Morio F , Scully J , Wolfe KH , Butler G . 2022. Systematic analysis of copy number variations in the pathogenic yeast Candida parapsilosis identifies a gene amplification in RTA3 that is associated with drug resistance. mBio 13:e0177722. doi:10.1128/mbio.01777-22 36121151 PMC9600344

[32] Burrack LS , Todd RT , Soisangwan N , Wiederhold NP , Selmecki A , Heitman J . 2022. Genomic diversity across Candida auris clinical isolates shapes rapid development of antifungal resistance in vitro and in vivo. mBio 13:e0084222. doi:10.1128/mbio.00842-22 35862787 PMC9426540

[33] Rhodes J , Beale MA , Vanhove M , Jarvis JN , Kannambath S , Simpson JA , Ryan A , Meintjes G , Harrison TS , Fisher MC , Bicanic T . 2017. A population genomics approach to assessing the genetic basis of within-host microevolution underlying recurrent cryptococcal meningitis infection. G3(Bethesda) 7:1165–1176. doi:10.1534/g3.116.037499 28188180 PMC5386865

[34] Bergström A , Simpson JT , Salinas F , Barré B , Parts L , Zia A , Nguyen Ba AN , Moses AM , Louis EJ , Mustonen V , Warringer J , Durbin R , Liti G . 2014. A high-definition view of functional genetic variation from natural yeast genomes. Mol Biol Evol 31:872–888. doi:10.1093/molbev/msu037 24425782 PMC3969562

[35] Chow EWL , Morrow CA , Djordjevic JT , Wood IA , Fraser JA . 2012. Microevolution of Cryptococcus neoformans driven by massive tandem gene amplification. Mol Biol Evol 29:1987–2000. doi:10.1093/molbev/mss066 22334577

[36] Zhai B , Ola M , Rolling T , Tosini NL , Joshowitz S , Littmann ER , Amoretti LA , Fontana E , Wright RJ , Miranda E , Veelken CA , Morjaria SM , Peled JU , van den Brink MRM , Babady NE , Butler G , Taur Y , Hohl TM . 2020. High-resolution mycobiota analysis reveals dynamic intestinal translocation preceding invasive candidiasis. Nat Med 26:59–64. doi:10.1038/s41591-019-0709-7 31907459 PMC7005909

[37] West PT , Peters SL , Olm MR , Yu FB , Gause H , Lou YC , Firek BA , Baker R , Johnson AD , Morowitz MJ , Hettich RL , Banfield JF . 2021. Genetic and behavioral adaptation of Candida parapsilosis to the microbiome of hospitalized infants revealed by in situ genomics, transcriptomics, and proteomics. Microbiome 9:142. doi:10.1186/s40168-021-01085-y 34154658 PMC8215838

[38] Moore IK , Martin MP , Dorsey MJ , Paquin CE . 2000. Formation of circular amplifications in Saccharomyces cerevisiae by a breakage-fusion-bridge mechanism. Environ Mol Mutagen 36:113–120. doi:10.1002/1098-2280(2000)36:2<113::aid-em5>3.0.co;2-t 11013409

[39] Møller HD , Parsons L , Jørgensen TS , Botstein D , Regenberg B . 2015. Extrachromosomal circular DNA is common in yeast. Proc Natl Acad Sci U S A 112:E3114–22. doi:10.1073/pnas.1508825112 26038577 PMC4475933

[40] Srivastava A , Sircaik S , Husain F , Thomas E , Ror S , Rastogi S , Alim D , Bapat P , Andes DR , Nobile CJ , Panwar SL . 2017. Distinct roles of the 7-transmembrane receptor protein RTA3 in regulating the asymmetric distribution of phosphatidylcholine across the plasma membrane and biofilm formation in Candida albicans. Cell Microbiol 19. doi:10.1111/cmi.12767 PMC5720375 28745020

[41] Whaley SG , Tsao S , Weber S , Zhang Q , Barker KS , Raymond M , Rogers PD . 2016. The RTA3 gene, encoding a putative lipid translocase, influences the susceptibility of Candida albicans to fluconazole. Antimicrob Agents Chemother 60:6060–6066. doi:10.1128/AAC.00732-16 27480868 PMC5038240

[42] Widmer F , Wright LC , Obando D , Handke R , Ganendren R , Ellis DH , Sorrell TC . 2006. Hexadecylphosphocholine (miltefosine) has broad-spectrum fungicidal activity and is efficacious in a mouse model of cryptococcosis. Antimicrob Agents Chemother 50:414–421. doi:10.1128/AAC.50.2.414-421.2006 16436691 PMC1366877

[43] Vila T , Ishida K , Seabra SH , Rozental S . 2016. Miltefosine inhibits Candida albicans and non-albicans Candida spp. biofilms and impairs the dispersion of infectious cells. Int J Antimicrob Agents 48:512–520. doi:10.1016/j.ijantimicag.2016.07.022 27667564

[44] Lauer S , Avecilla G , Spealman P , Sethia G , Brandt N , Levy SF , Gresham D . 2018. Single-cell copy number variant detection reveals the dynamics and diversity of adaptation. PLoS Biol 16:e3000069. doi:10.1371/journal.pbio.3000069 30562346 PMC6298651

[45] Avecilla G , Chuong JN , Li F , Sherlock G , Gresham D , Ram Y . 2022. Neural networks enable efficient and accurate simulation-based inference of evolutionary parameters from adaptation dynamics. PLoS Biol 20:e3001633. doi:10.1371/journal.pbio.3001633 35622868 PMC9140244

